# Ultrasonic extraction, structural characterization, and antioxidant activity of oligosaccharides from red yeast rice

**DOI:** 10.1002/fsn3.2660

**Published:** 2021-11-24

**Authors:** Kai Liu, Long Xie, Huan Gu, Jia Luo, Xiaofang Li

**Affiliations:** ^1^ School of Pharmacy Chengdu University of Traditional Chinese Medicine Chengdu China

**Keywords:** oligosaccharide, red yeast rice, structure elucidation, ultrasonic‐assisted extraction

## Abstract

Red yeast rice is consumed as a medicinal food to lower blood lipids. Besides, it is used to color food, make wine, etc. In this study, water‐soluble oligosaccharides in red yeast rice were extracted by ultrasonic‐assisted extraction method. The parameters to extract oligosaccharides from red yeast rice were optimized by the Box‐Behnken design under the following optimal extraction conditions: extraction temperature, 60°C; extraction time, 97 min; and liquid/material ratio, 25 ml/g. The structure and the antioxidant activity of the new oligosaccharide were preliminarily investigated. Total carbohydrates extracted from red yeast rice with 80% ethanol–water solution (v/v) were first removed from pigments using D101 macroporous adsorption resin. The total sugar contents were then purified by DE52 resins and Sephadex G‐25 resins to obtain red yeast rice oligosaccharides, coded as RYRO1. Structural characterization experiments indicated that RYRO1 is an oligosaccharide with a weight average molecular weight of 874 Da and a theoretical degree of polymerization of 4.86. RYRO1 is composed of mannose, glucosamine, glucose, and galactose with a molar ratio of 0.248:0.019:1:0.026. The ABTS, DPPH, and hydroxyl free radical scavenging assays showed antioxidant nature of RYRO1.

## Graphical abstract

A new oligosaccharide was isolated from red yeast rice. RYRO1 consists of mannose, glucosamine, glucose, and galactose. RYRO1 possesses in vitro antioxidant activity.

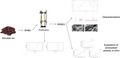

## 1 INTRODUCTION

Red yeast rice (RYR) is produced from rice through fermentation by *Monascus purpureus* (Cicero et al., 2021; Luo et al., 2020). In East Asia, red yeast rice is a widely used food additive. It is also utilized to produce alcoholic beverages and fermented foods in China, Korea, and Japan. In addition, RYR is also considered health food. According to statistics, there are more than 100 kinds of health food containing red yeast rice in China (Zhu et al., 2019). Modern pharmacological studies have proved that RYR exerts protective effects on the liver, the pancreas, blood vessels, and other organs (Hu et al., 2020). The main ingredients of RYR are *Monascus* pigments and monacolins (Hu et al., 2020), and monacolin K, the prime component of monacolins, has been utilized as a lipid‐lowering agent (Xiong et al., 2019).

Oligosaccharides are any carbohydrates consisting between two and ten monosaccharides connected by either an alpha‐ or beta‐glycosidic link. Oligosaccharides extracted from natural resources are proved to possess a wide variety of bioactivities. Bai et al. extracted an oligosaccharide with the average relative molecular weight of 318 Da from *Codonopsis pilosula* and found it could increase the secretion of cytokines (TNF‐α, NO, etc.) by triggering the MAPK signaling pathway (Bai et al., 2020). Lan et al. demonstrated that chitosan oligosaccharides are capable of maintaining intestinal integrity under oxidative stress by modulating the intestinal oxidative status and the release of inflammatory cytokines (Lan et al., 2021). Yu et al. extracted three novel oligosaccharides from Kunlun chrysanthemum flower tea and proved their inhibitory effects on α‐amylase and α‐glucosidase (Yu et al., 2021). What's more, oligosaccharides derived from fungi have attracted the interest of researchers. For example, some oligosaccharides composed of mannose were extracted from *Tremella fuciformis* Berk. They were characterized as (1 → 3)‐mannan oligosaccharides with a straight chain. Furthermore, an in vitro study demonstrated some of them possessed cytokine‐stimulating activity (Gao et al., 2020). Ding et al. (2015) isolated one major oligosaccharide composed of D‐glucose and D‐xylose from *Lactarius deliciosus* (L. ex Fr.) Gray and in vivo studies demonstrated that it possessed antitumor activity. Moreover, heptasaccharides derived from the basidiomycete *Trametes versicolor* showed significant effects on mycotoxin inhibition in *A*. *flavus* and *A. carbonarius* (Loncar et al., 2021).

RYR is a fermented product; in our previous in vivo study indicated that, the 3.49‐kDa nonstarch polysaccharides were isolated from RYR and that it possessed the gastrointestinal‐protective effect (Luo et al., 2020). Most of the modern research into RYR focused on small molecule substances such as statins and pigments; yet, oligosaccharides extracted from medicinal foods have been proved to have many beneficial effects, which provides more evidence for further development of these substances. However, the studies on the structural characterization and bioactivity of oligosaccharides in RYR are limited. Hence, studies on the content, structural information, and bioactivity of oligosaccharides in RYR are essential.

**FIGURE 1:**
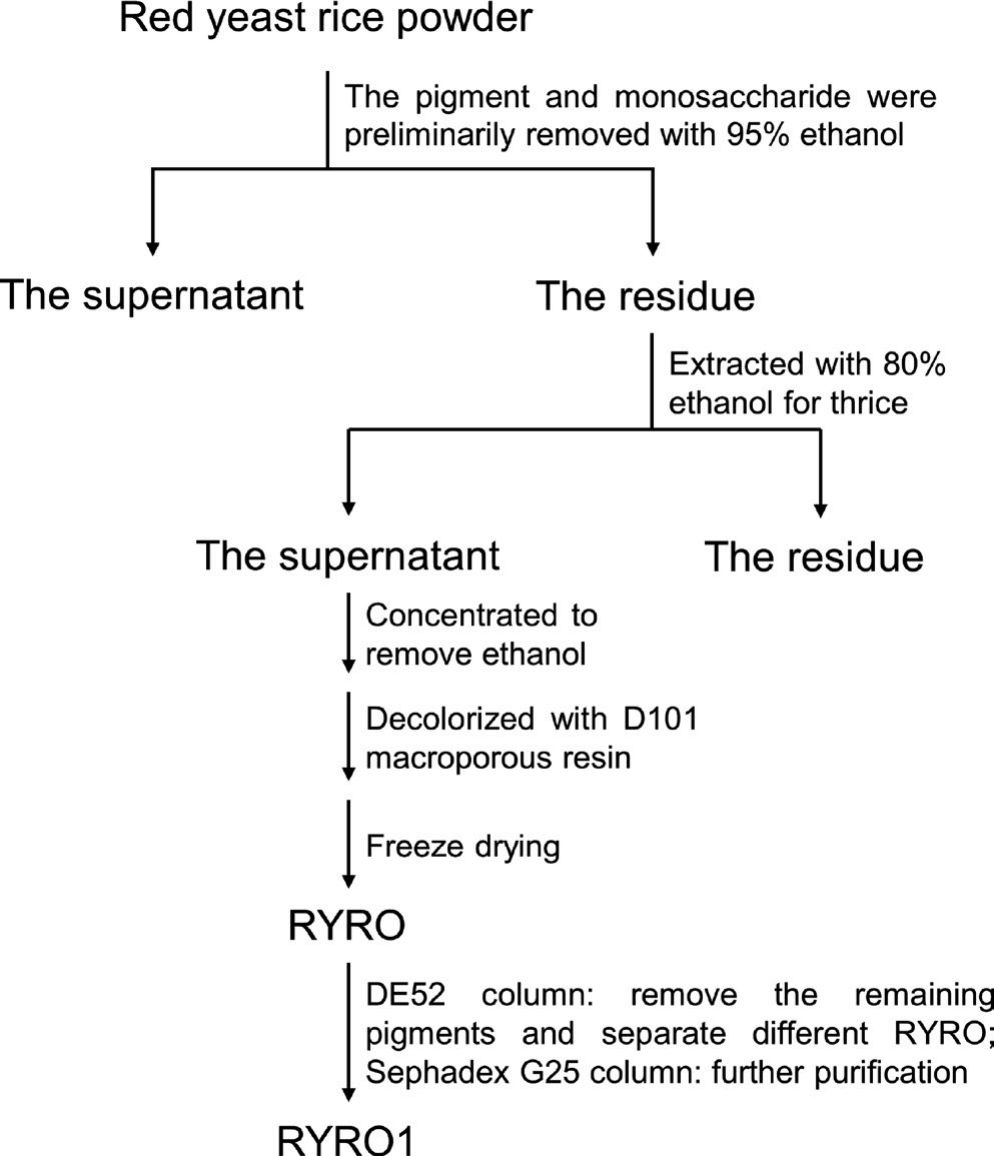
Flowchart showing isolation and purification of RYRO1 from red yeast rice

Here, RYR oligosaccharides were isolated and purified from RYR, and the extraction flow chart is shown in Figure 1. Subsequently, precolumn derivatization HPLC, UPLC‐TOF‐MS, HPGPC, FT‐IR, NMR, and *SEM* were employed to elucidate the structure of RYRO1, and finally, its antioxidative activity was investigated by DPPH, ABTS, and hydroxyl free radical assays.

## 2 MATERIALS AND METHODS

### 2.1 Materials and reagents

RYR was purchased from Sichuan Sino‐Dandard Pharmaceutical Co. (Mianyang, Sichuan, China). Monosaccharide standards D‐glucose (Glu), D‐mannose (Man), D‐galactose (Gal), and D‐glucosamine (GlcN) were obtained from Chengdu MUST Bio‐Technology Co., Ltd. (Chengdu, Sichuan, China). Trifluoroacetic acid (TFA) and 1‐phenyl‐3‐ methyl‐5‐pyrazolone (PMP) were purchased from the SinoPharm Chemical Reagents Co. (Shanghai, China). Sodium tetraborate and m‐hydroxybiphenyl were obtained from Sigma Aldrich (St. Louis, MO, USA). Dialysis bags (molecular cutoff of 500 Da) were obtained from Spectrum Chemical Mfg. Corp. (New Brunswick, NJ, USA). Deionized water was obtained using an Ultrapure system (Chengdu, Sichuan, China). 1,1‐Diphenyl‐2‐picryl‐hydrazyl (DPPH) and 2,2‐azino‐bis‐3‐ethylbenzothiazoline‐6‐sulfonic acid (ABTS) were obtained from Sigma‐Aldrich. Hydroxyl radical assay kits were obtained from Nanjing Jiancheng Bioengineering Institute (Nanjing, China). HPLC‐grade acetonitrile was purchased from Fisher Chemical (Waltham, MA, USA). All other chemicals were of analytical grade.

### 2.2 Ultrasonic‐assisted extraction of oligosaccharides from RYR

Oligosaccharides were extracted from RYR by ultrasonic‐assisted extraction. The dried red yeast rice was ground into a fine powder and was prepared for use. An appropriate amount of RYR fine powder was placed in a conical flask, and 95% ethanol solution was added at a liquid/material ratio of 20 ml/g and then sealed. The sample was then shaken at 150 rpm for 30 min at 40°C to initially remove the pigments and monosaccharides. Finally, the sample was filtered under reduced pressure and the filter residue was collected. The above procedure was repeated three times. The sample (3.0 g) was extracted with 80% ethanol solution using a 40‐kHz ultrasonic cleaner (KQ5200DE; Kunshan Ultrasonic Co., Kunshan, China) equipped with a digital timer and temperature controller, and a single‐factor experimental design was performed under the following conditions: liquid/material ratio (10–30 ml/g), extraction time (30–180 Min), and extraction temperature (25–60°C). When optimizing factors in each experiment, one factor was changed while the other factors were kept constant. After extraction, the supernatant was obtained by centrifugation (L‐550; Xiangyi Centrifuge Instrument Co., Changsha, China) and filtration under reduced pressure. Each sample was extracted three times and the filtrates were combined. The content of oligosaccharides obtained from red yeast rice was determined by the phenol sulfuric acid method (Zhu et al., 2020). The yield of oligosaccharides from red yeast rice was calculated by [oligosaccharide mass]/[sample mass] ×100%.

### 2.3 Optimization of the extraction conditions by the Box–Behnken design

Based on the preliminary single‐factor experimental results, a three‐level, three‐factor Box–Behnken design method was used to optimize the study by Design‐Expert software. Liquid/material ratio (*X*_1_; 20, 25, and 30 ml/g), extraction time (*X*_2_; 60, 90, and 120 min), and extraction temperature (*X*_3_; 50, 60, and 70°C) were the independent variables to be optimized for the extraction of the RYR oligosaccharides (RYRO). The yield of oligosaccharide (%) (Y) was employed as the response of the design experiment. Based on the Box–Behnken design experimental data, a second‐order polynomial equation was utilized to describe the relationship between the predicted response and the variables as follows:

$$Y=\beta_{0}+\sum \beta_{i}x_{i}+\sum \beta_{\mathrm{ii}}x_{i}^{2}+\sum \beta_{\mathrm{ij}}x_{i}x_{j}$$

where Y is the predicted response, *x_i_* and *x_j_* are the levels of the independent variables, and *β*_0_, *β_i_*, *β_ii_*, and *β_ij_* are the intercept, linear coefficient, quadratic coefficient, and interaction coefficient of the model, respectively.

### 2.4 Purification and structure characterization of RYRO1

#### 2.4.1 Purification of RYRO

Ten grams of RYR was crushed and ultrasonically (40 kHz, 200W) extracted thrice with 250 ml of 80% ethanol–water solution under 40°C for 2 hr. The extracts were combined and concentrated under reduced pressure (40°C, −0.1 Mpa) to remove ethanol, and the volume was made up to 400 ml with deionized water. Twenty grams of activated D101 macroporous adsorption resin and 400 ml of the extract were placed in an Erlenmeyer flask, decolorized in an air shaker at 150 rpm, 40°C for 2 hr, and then filtered and lyophilized for future studies.

**FIGURE 2:**
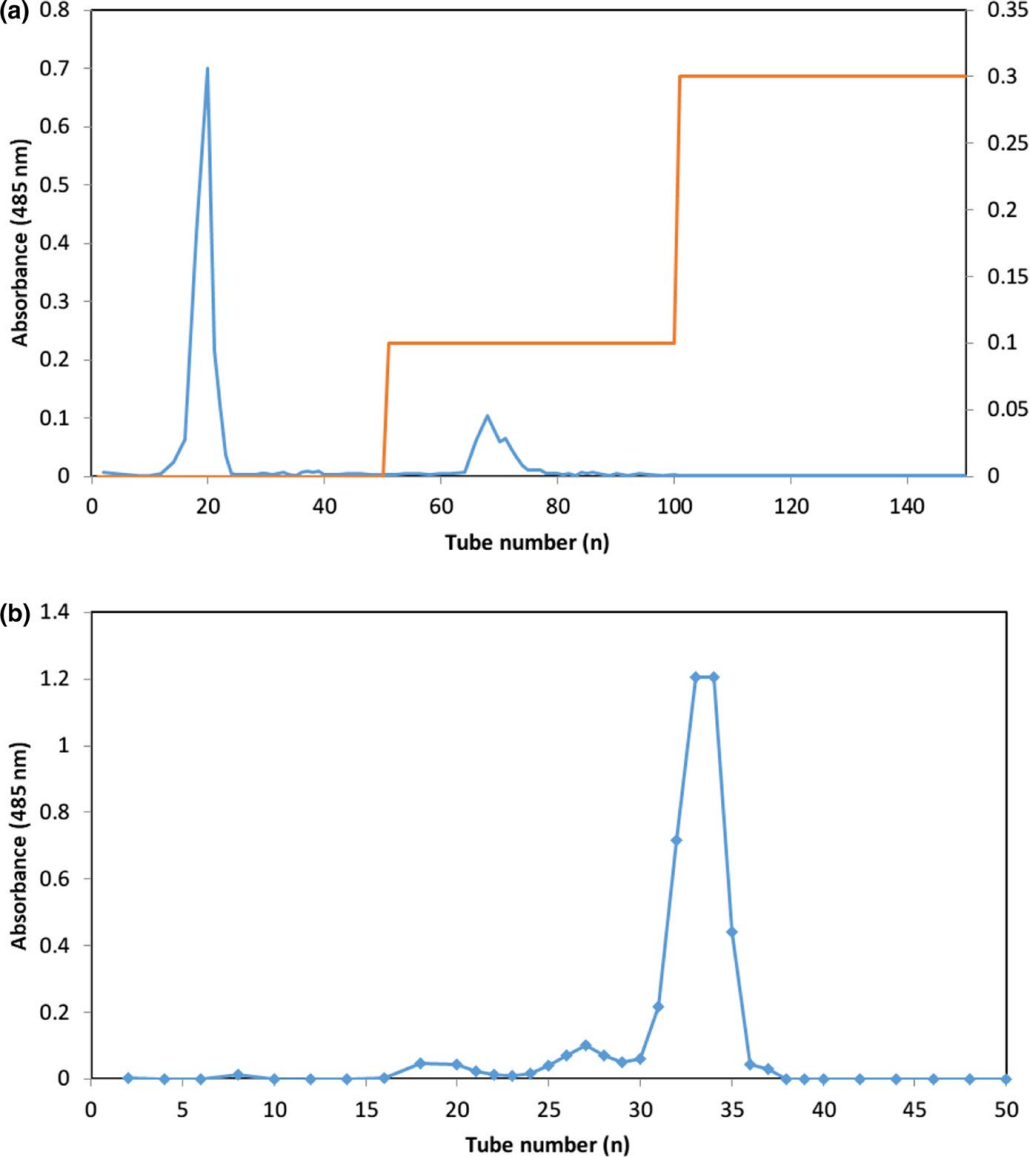
(a) Elution profile of RYRO on a DEAE column with deionized water, 0.1 M and 0.3 M NaCl solution; (b) elution profile of RYRO1 on a Sephadex G‐25 gel chromatography column with deionized water

For subsequent studies, RYRO was purified using an ion‐exchange chromatography column and size exclusion column. After elution, the total sugar contents were determined by the phenol sulfuric acid method and the content of uronic acid was determined by the sulfamate/meta‐hydroxydiphenyl assay using glucuronic acid as the standard (Blumenkrantz & Asboe‐Hansen, 1973). The RYRO1 aqueous solution was scanned with a UV‐VIS spectrophotometer in the range 200–400 nm to determine whether it contained protein and nucleic acid. RYRO was dissolved in 8 ml deionized water and then purified with a DEAE (Biobying, Beijing, China) column (2.8 × 40 cm, i.d.) that had been equilibrated using deionized water. After loading, elution was performed with deionized water, 0.1 M, and 0.3 M NaCl solution at a flow rate of 0.8 ml/min, and fractions were collected with an automatic fraction collector (8 ml/tube). The elution profile is shown in Figure 2a. The first aqueous fraction was named RYRO1 and collected, lyophilized, and dissolved in 3 ml deionized water, and loaded onto a Sephadex G‐25 (H&E, Beijing, China) column (1.8 × 50 cm, i.d.) preequilibrated with water. After loading, the size exclusion column was eluted with deionized water at a rate of 0.3 ml/min, and 3 ml of the eluate was collected per tube. The elution profile is shown in Figure 2b. The collected purified fraction was concentrated and lyophilized for further study.

#### 2.4.2 Determination of monosaccharide composition of RYRO1

1) Hydrolysis procedures for RYRO1

Monosaccharides composition analysis was performed with reference to the method reported by Zhao et al. with minor modifications (Zhao et al., 2020). Briefly, saccharide samples (40 mg) were mixed with 8 ml TFA (2 mol/L) in a stoppered colorimetric tube, sealed and placed in an oven at 110°C for 5 hr hydrolysis. The mixture was then cooled to room temperature, and TFA was evaporated under reduced pressure. After adding methanol and evaporating under reduced pressure six times to remove residual TFA, the obtained monosaccharides were dissolved with 5 ml deionized water.

2) Precolumn derivatization of mixed monosaccharide standards and samples

A volume of 0.2 ml of a mixed standard solution (2 mM) containing glucose, glucosamine, mannose, and galactose, and 0.2 ml of the RYRO1 hydrolysate were mixed in an EP tube, and an isometric PMP–methanol solution (0.5 mol/L) and NaOH solution (0.3 mol/L) were sequentially added. The mixture was incubated at 70°C in a water bath for 1 hr, cooled to room temperature, and neutralized with HCl solution (0.3 mol/L). One milliliter of chloroform was added and shaken for 1 min, and the chloroform layer was discarded. The extraction process was repeated three times. Finally, the aqueous layer was supplemented to 5 ml with deionized water, and 1 ml was filtered through a 0.45‐μm microporous membrane prior to further HPLC analysis.

3) HPLC analysis

PMP derivatives of monosaccharides were analyzed by an Agilent 1260 HPLC system (Agilent, Santa Clara, CA, USA) equipped with a CAPCELL PAK C18 column (4.6 mm i.d. × 250 mm, 5 μm), and detected by a UV‐Vis DAD detector. Ten microliters of PMP derivatives was injected and eluted with 82.0% phosphate buffer (0.05 M, pH 6.8) and 18.0% acetonitrile (v/v) at a flow rate of 0.8 ml/min at 30°C with UV detection at 245 nm.

#### 2.4.3 Ultra‐performance liquid chromatography coupled with time‐of‐flight mass spectrometry (UPLC‐TOF‐MS) analysis

RYRO1 was dissolved in H_2_O for UPLC‐TOF‐MS analysis. The experiment was conducted on a Waters SYNAPT XS high‐resolution mass spectrometer (Milford, MA, USA). Mass spectrometry data were analyzed and processed by Waters Masslynx^®^ software.

#### 2.4.4 Determination of molecular weight of RYRO1

The molecular weight distribution of RYRO1 was determined by high‐performance gel permeation chromatography (HPGPC) employing an Agilent Infinity 1260 system, equipped with a refractive index detector (Agilent, California, USA). The tested sample (5 mg/ml) was filtered through a 0.45‐μm polyether sulfone filter before injecting into the system. The columns in series were two PL aquagel‐OH MIXED‐H (7.5 mm×30 cm, 8 μm; Agilent, California, USA), and the column temperature was 30°C. The mobile phase was 0.15 M NaNO_3_–water solution with a flow rate of 1 ml/min. The total analysis time was 30 min. To calculate the relative molecular mass of RYRO1, the standard curves with retention time and log Mw as horizontal and vertical coordinates were established using Dextran Standards with number averaging Mw of 194, 610, 1450, 3860, 7920, 16,100, 28,300, 49,640, 66,200, 117,900, 545,000, and 1,046,000 Da, respectively.

#### 2.4.5 Fourier transform infrared (FT‐IR) spectral analysis

FT‐IR spectra of RYRO1 were obtained on a Thermo Scientific spectrometer (Thermo Scientific Technologies, CA, USA), equipped with a DTGS KBr detector to investigate the functional groups of RYRO1. Samples were mixed with spectroscopic grade KBr powder, ground, and pressed into 1‐mm slices. With distilled water as background subtraction, spectra were recorded in the range 4000–400 cm^‐1^ with a resolution of 4 cm^‐1^, and a total of 32 scans were performed.

#### 2.4.6 Nuclear magnetic resonance (NMR) analysis

Ten milligrams of RYRO1 was dissolved in 0.55 ml D_2_O (99.9%). ^1^H‐ and ^13^C‐NMR spectra of RYRO1 were recorded employing a Bruker AV NEO 600 MHz NMR instrument (Billerica, MA, USA). Chemical shifts were expressed in ppm.

#### 2.4.7 Scanning electron microscopy (SEM) analysis

RYRO1 was completely lyophilized, then a thin layer of gold was sprayed on its surface, followed by observation of its microstructure (100–5000 fold) with a Zeiss Supra 40 (Zeiss, Berlin, Germany) SEM under high vacuum conditions at a 10.00 kV acceleration voltage.

### 2.5 Antioxidant activity of RYRO1 in vitro

#### 2.5.1 DPPH radical scavenging assay

The DPPH scavenging ability of RYRO1 was measured by a previously reported method with minor modifications (Zhao et al., 2020). Various concentrations of RYRO1 (0.025–1.5 mg/ml) were added to isometric DPPH solution (0.1 mM), mixed well, and incubated in the dark for 30 min. The absorbance of each sample was read using a UV‐visible light spectrophotometer (Mapada Instruments, Shanghai, China), and the ability of the sample to scavenge DPPH radicals was calculated using the following equation:

$$\text{DPPH scavenging rate}\;\left(\%\right)=\left[1‐\frac{A_{i}‐A_{j}}{A_{0}}\right]\times 100\%$$

where *A_i_* is the absorbance of equal amounts of RYRO1 and DPPH, and *A_j_* is the absorbance value of equal amounts of RYRO1 and ethanol. The absorbance values of equal amounts of DPPH and ethanol were recorded as *A*_0_. Ascorbic acid was used as a positive control. The volume of each solution in the experiment was 1 ml.

#### 2.5.2 ABTS radical scavenging assay

The ABTS radical scavenging ability of RYRO1 was evaluated by a previously reported method with minor modifications (Zhao et al., 2020). The ABTS radical cation solution (ABTS•+) was prepared by reacting 7 mM solution and 2.45 mM potassium persulfate solution (1:1, v/v) at 25 degrees C for 14 hr in the dark. For use, the solution obtained from the reaction was diluted with ethanol to an absorbance of 0.700 ± 0.02 at 734 nm. Various concentrations of RYRO1 samples (30 μL) were added to 170 μL of ABTS•+ solution. After 6 min of reaction at room temperature, the absorbance of the above solution at 750 nm was recorded as *A_i_* using an enzyme marker, and the absorbance of the control containing equal amounts of ABTS•+ solution and ethanol was recorded as *A_0_*. The absorbance of 30 μL of sample and 170 μL of ethanol was recorded as *A_j_*. Using ascorbic acid as a positive control, the ABTS radical scavenging capacity was calculated using the following equation:

$$\text{ABTS radical scavenging rate}\;\left(\%\right)=\left[1‐\frac{A_{i}‐A_{j}}{A_{0}}\right]\times 100\%$$

#### 2.5.3 Hydroxyl free radical scavenging assay

The hydroxyl radical scavenging activity of different concentrations of RYRO1 (0.025–0.50 mg/ml) was measured according to the instructions provided in the hydroxyl radical assay kit. Ascorbic acid was employed as a positive control.

## 3 RESULTS AND DISCUSSION

### 3.1 Single‐factor experiment analysis

**FIGURE 3:**
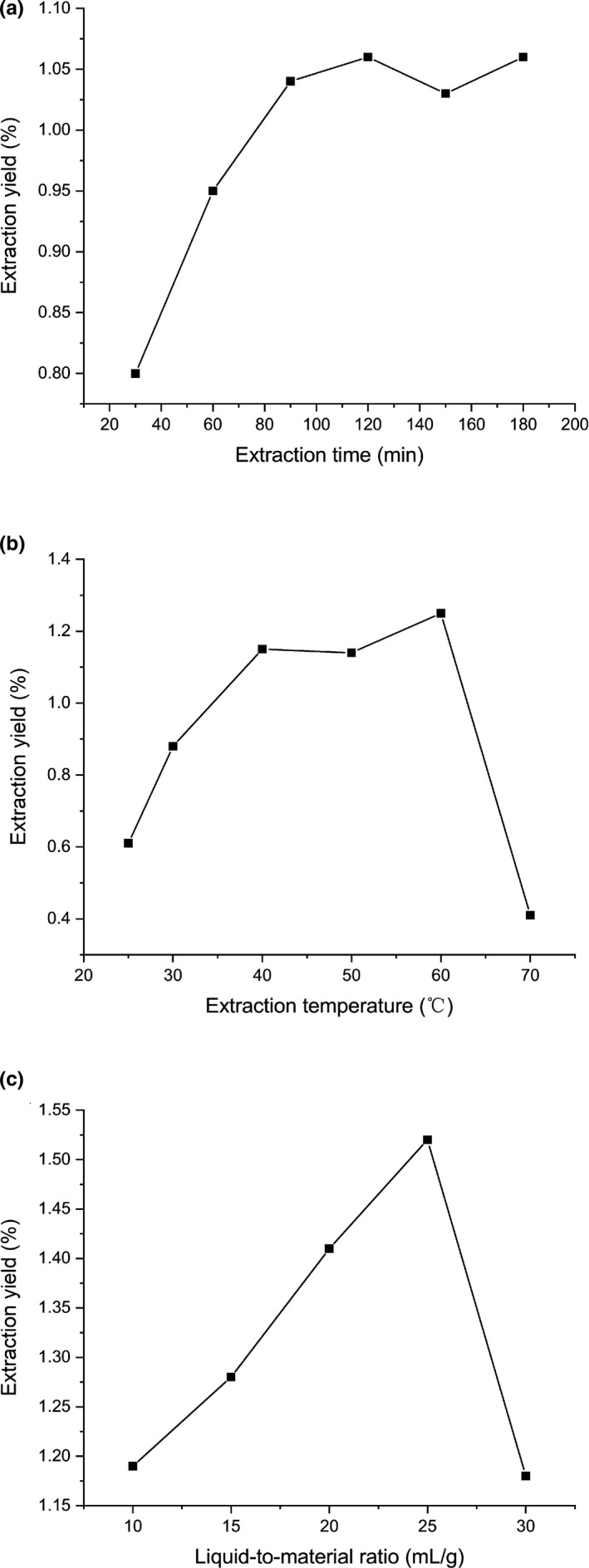
Effects of different extraction parameters on the yield of RYRO: (a) extraction time; (b) extraction temperature; and (c) liquid/material ratio

The effect of single factors (liquid/material ratio, extraction time, and extraction temperature) on the extraction yield of oligosaccharides is shown in Figure 3.

According to Figure 3a, when the extraction temperature was 40°C and the liquid/material ratio was 20 ml/g, the yield of oligosaccharides increased remarkably from 30 to 90 min. However, the yield stopped rising when the extraction time extended due to the extraction equilibrium (Liu et al., 2019). Hence, 90 min was selected as the center point for the response surface methodology based on the Box–Behnken design. As can be seen from Figure 3b, when the extraction time was 90 min and the liquid/material ratio was 20 ml/g, the extraction yield increased significantly at the temperature between 25 and 40°C. However, when the temperature was between 40 and 60°C, the increase in yield was not significant. However, when the temperature reached 70°C, the yield dropped sharply. This may be due to the degradation of oligosaccharides caused by high temperatures (Gu et al., 2021). Therefore, an extraction temperature of 60°C was selected as the center point for the following optimization. Based on Figure 3c, when the extraction temperature of 60°C and extraction time was 90 min, the extraction rate of oligosaccharides increased significantly when the liquid/material ratio increased from 10 to 25 ml/g but decreased significantly when the liquid/material ratio increased from 25 to 30 ml/g. This may be due to the tendency of the red yeast rice powder to clump together at low ratios, which makes it difficult for water to enter, while higher liquid/material ratios result in lower concentrations and viscosity of the extractant, leading to the dissolution of oligosaccharides in water (Nuerxiati et al., 2019). Therefore, the liquid/material ratio of 25 ml/g was selected for the following optimization.

### 3.2 Optimization of the extraction conditions of RYRO

Based on the results of single‐factor experiments, the response surface methodology was applied to optimize the extraction of oligosaccharides. The design and data of the 17‐run experiments are shown in Table 1. The following quadratic polyatomic regression equation was established:

$$\begin{array}{c} Y=‐12.634\;+\;0.4655X_{1}+\;0.061017X_{2}+\;0.17435X_{3}‐\;0.000117X_{1}X_{2}‐0.00055X_{1}X_{3}‐\;\\ 0.000042X_{2}X_{3}‐\;0.00837X_{1}^{2}‐\;0.000277X_{2}^{2}‐\;0.001293X_{3}^{2} \end{array}$$

where *Y* is the extraction yield of RYRO and *X_1_, X_2_,* and *X_3_* represented liquid/material ratio, extraction time, and extraction temperature, respectively.

**TABLE 1:** Box–Behnken design and observed responses

Run	Liquid/material ratio (ml/g)	Extraction time (min)	Extraction temperature (°C)	Extraction yield (%)
1	0	0	0	1.52
2	1	0	−1	1.21
3	−1	1	0	1.25
4	0	0	0	1.53
5	1	−1	0	0.90
6	−1	0	1	1.20
7	−1	0	−1	1.10
8	0	0	0	1.52
9	0	0	0	1.50
10	0	0	0	1.51
11	1	1	0	1.23
12	1	0	1	1.20
13	0	1	−1	1.30
14	0	−1	−1	0.95
15	−1	−1	0	0.85
16	0	1	1	1.30
17	0	−1	1	1.00

The ANOVA was used to assess the significance and fitness of the regression models, and the results are shown in Table 2.

**TABLE 2:** ANOVA for the response surface quadratic model

Source	Sum of squares	Degree of freedom	Mean of squares	*F*‐Value	*p*‐value	Significant level
Model	0.8196	9	0.0911	274.76	<.0001	**
*X*_1_	0.0025	1	0.0025	7.39	.0298	*
*X*_2_	0.2381	1	0.2381	718.25	<.0001	**
*X*_3_	0.0024	1	0.0024	7.39	.0298	*
*X*_1_ *X_2_*	0.0012	1	0.0012	3.70	.0960	
*X*_1_ *X*_3_	0.0030	1	0.0030	9.13	.0194	*
*X*_2_ *X*_3_	0.0006	1	0.0006	1.89	.2120	
*X*_1_^2^	0.1844	1	0.1844	556.26	<.0001	**
*X*_2_^2^	0.2616	1	0.2616	789.25	<.0001	**
*X*_3_^2^	0.0703	1	0.0703	212.23	<.0001	**
Residual	0.0023	7	0.0003			
Lack of fit	0.0018	3	0.0006	4.62	.0868	
Pure error	0.0005	4	0.0001			
Cor total	0.8219	16				
*R* ^2^	0.9972					
*R* ^2^_Adj_	0.9935					

Note

*Significant differences (*p* < .05). ** Extremely significant differences (*p* < .01).

According to Table 2, the high F‐value (67.42) and low *p*‐value (*p* < .0001) indicate that the model is highly significant. The F and p values of lack of fit were 4.62 and 0.0868, respectively. The results showed that the model was able to predict the yield of RYRO. The values of *R* ^2^, adj‐*R* ^2^, and coefficient of variation were 0.9972, 0.9935, and 1.47, respectively, indicating that the model predicted the yield of RYRO accurately and reliably. The p‐value was utilized as a tool to test the significance of each coefficient. A smaller p‐value indicated that the corresponding coefficient was more significant. As shown in Table 2, the extraction yield of RYRO was significantly (*p* < .05) affected by the linear coefficients (*X*_1_, *X*_2,_, and *X*_3_) and the quadratic coefficients (*X*_1_^2^, *X*_2_^2^, *X*_3_^2^). However, the two interaction coefficients (*X*_1_ *X*_2_ and *X*_2_ *X*_3_) had less effect on the extraction yield of RYRO due to the higher p‐value (*p* >.05).

**FIGURE 4:**
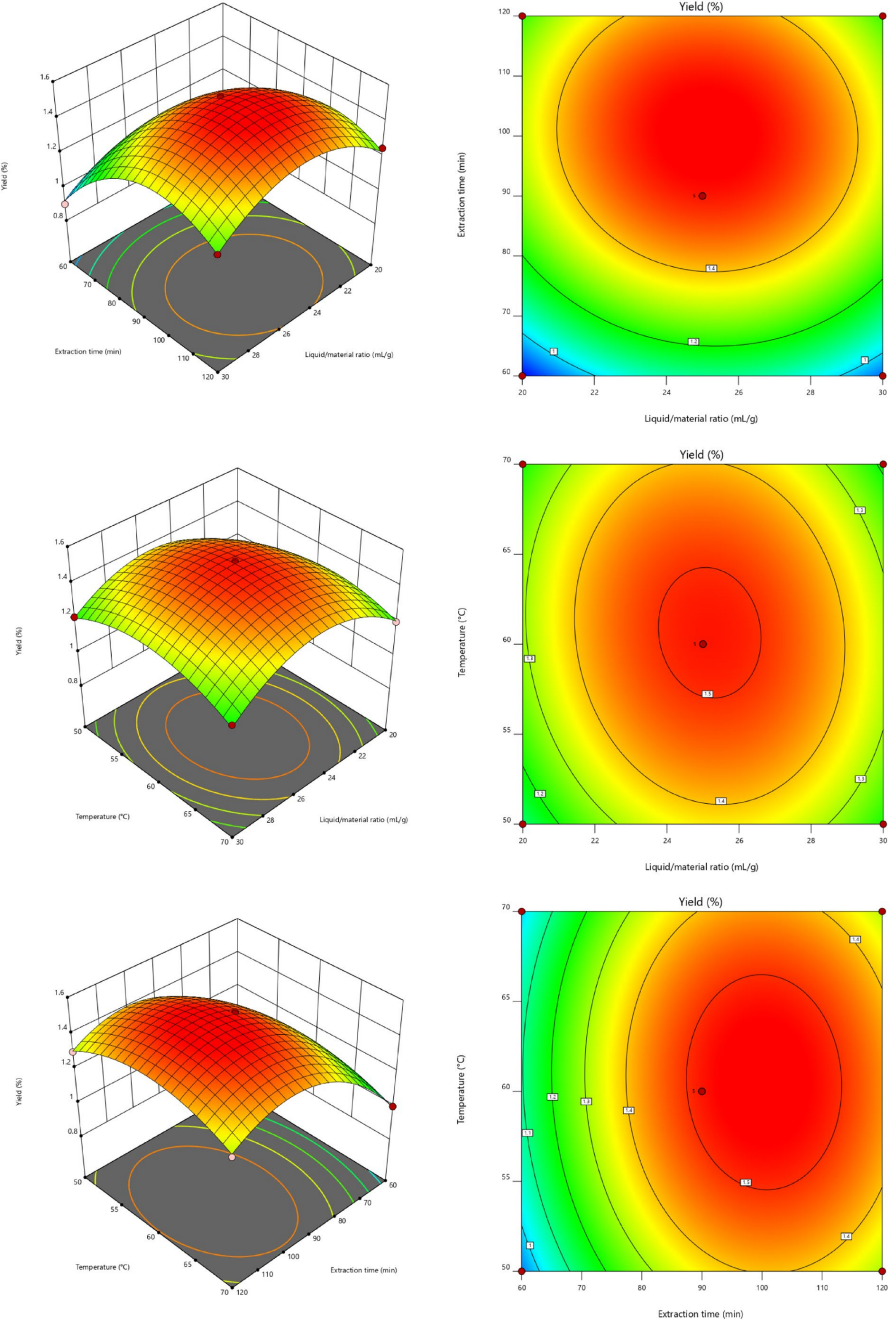
3D response surface and 2D contour plots showing the effects of liquid/material ratio (*X*_1_), extraction temperature (*X*_2_), and extraction temperature (*X*_3_) on the yield of RYRO

Three‐dimensional response surface plots and contour plots (Figure 4) can depict the interactions between the factors and the extraction yield of RYRO. Based on the results, the optimal extraction process for RYRO is as follows: extraction time of 96.7 min, liquid/material ratio of 24.96 ml/g, and extraction temperature of 61.94°C. The extraction rate was 1.540% under this optimal process. The optimal conditions were slightly modified: that is, the liquid‐to‐material ratio was 25 ml/g, the extraction time was 97 min, and the extraction temperature was 60°C. A validation experiment was performed under these conditions. The actual extraction rate of 1.52% was in good agreement with the predicted value, indicating that the process can be directly used to guide the extraction of RYRO.

### 3.3 Structure characterization

#### 3.3.1 General properties of RYRO1

**FIGURE 5:**
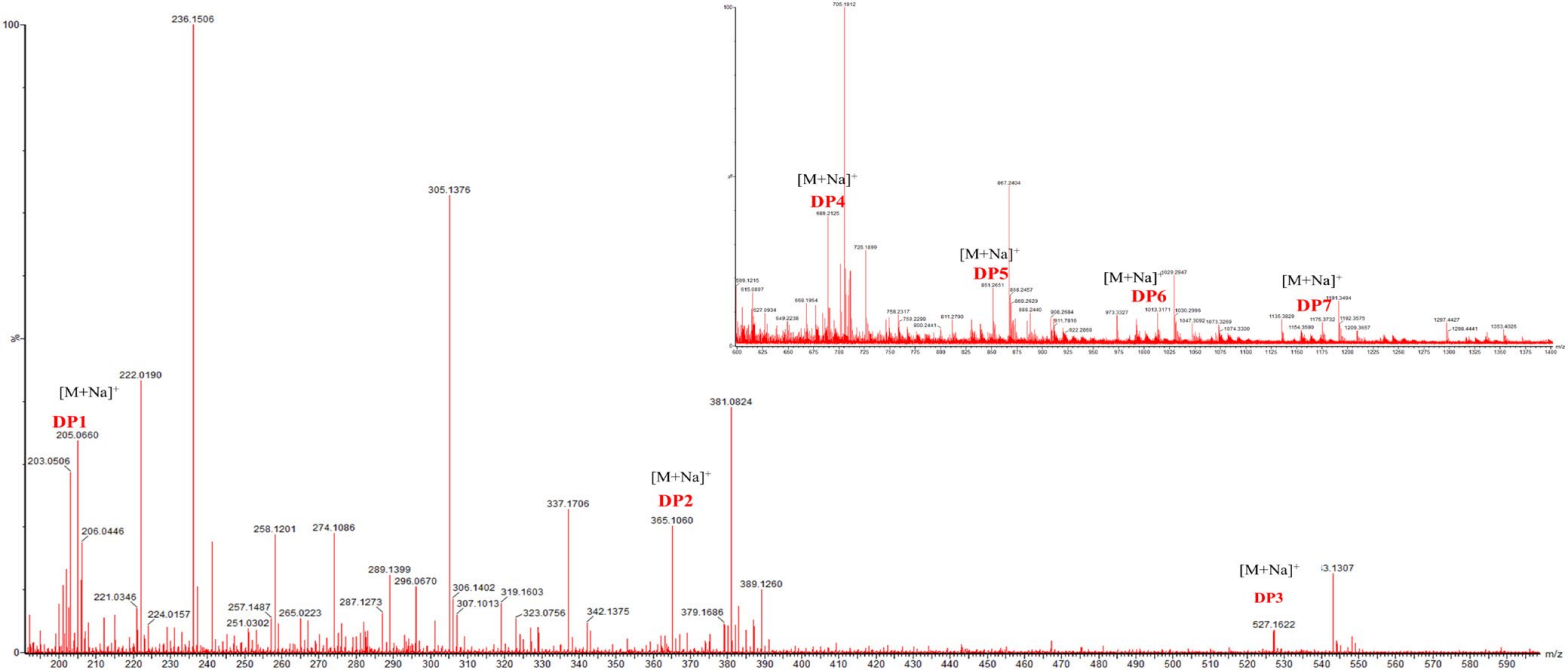
UPLC‐TOF‐MS spectrum of RYRO1

RYRO was obtained from RYR through ultrasonic‐assisted 80% ethanol extraction. Then, it was purified to obtain RYRO1. The sugar content was 93.7% and no uronic acid was detected. The UV–Vis spectrum showed no absorption in the range 200–400 nm, indicating that RYRO1 contains no nucleic acids or proteins (Qin et al., 2019). The results of UPLC‐TOF‐MS indicate that RYRO1 was mainly composed of monosaccharides, disaccharides, trisaccharides, and tetrasaccharides, and trace amounts of oligosaccharides with DP from 5 to 7 (Figure 5).

#### 3.3.2 Monosaccharide composition of RYRO1

**FIGURE 6:**
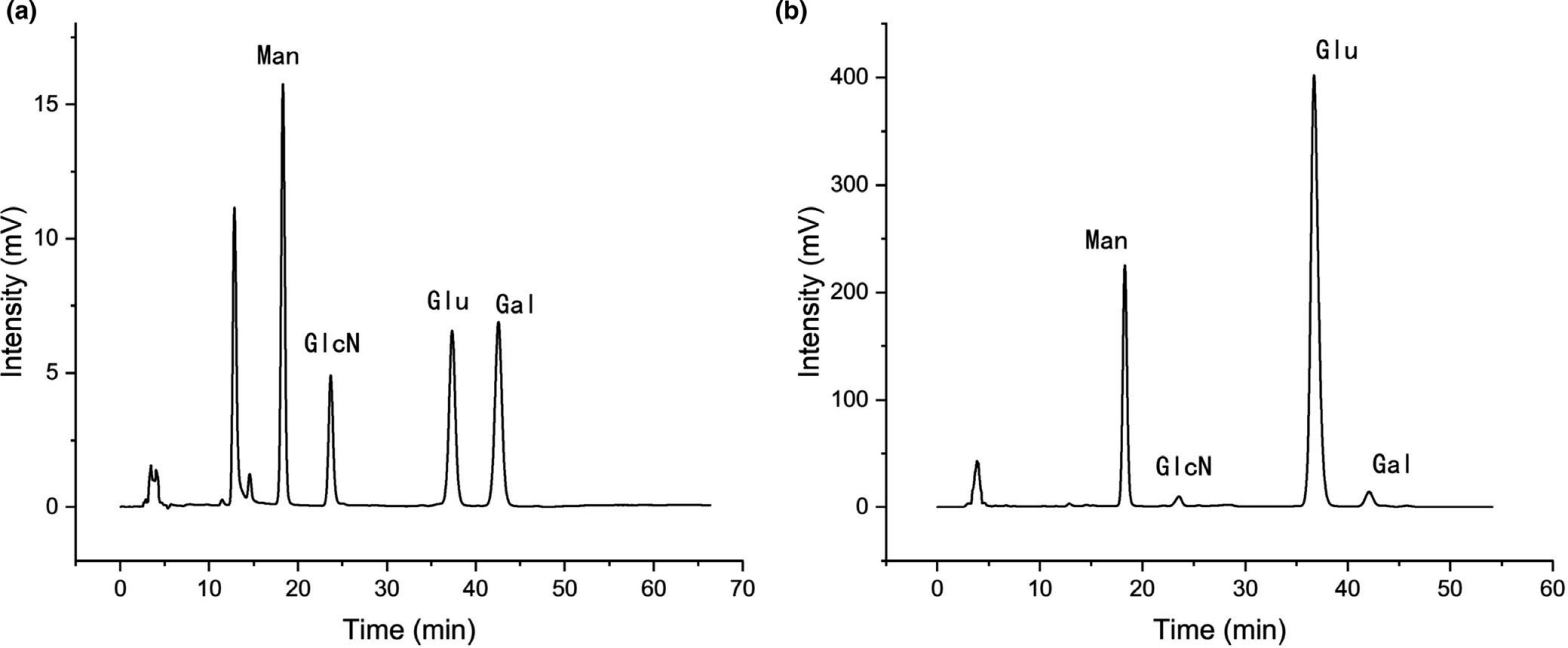
(a) HPLC chromatograms of standard monosaccharides; (b) monosaccharide composition of RYRO1

RYRO1 was purified employing a DEAE column and a size‐exclusion column (Sephadex G‐25). Precolumn derivatization HPLC showed that RYRO1 is composed of mannose, glucosamine, glucose, and galactose with a molar ratio of 0.248:0.019:1:0.026 (Figure 6a,b).

#### 3.3.3 Determination of molecular weight of RYRO1

**FIGURE 7:**
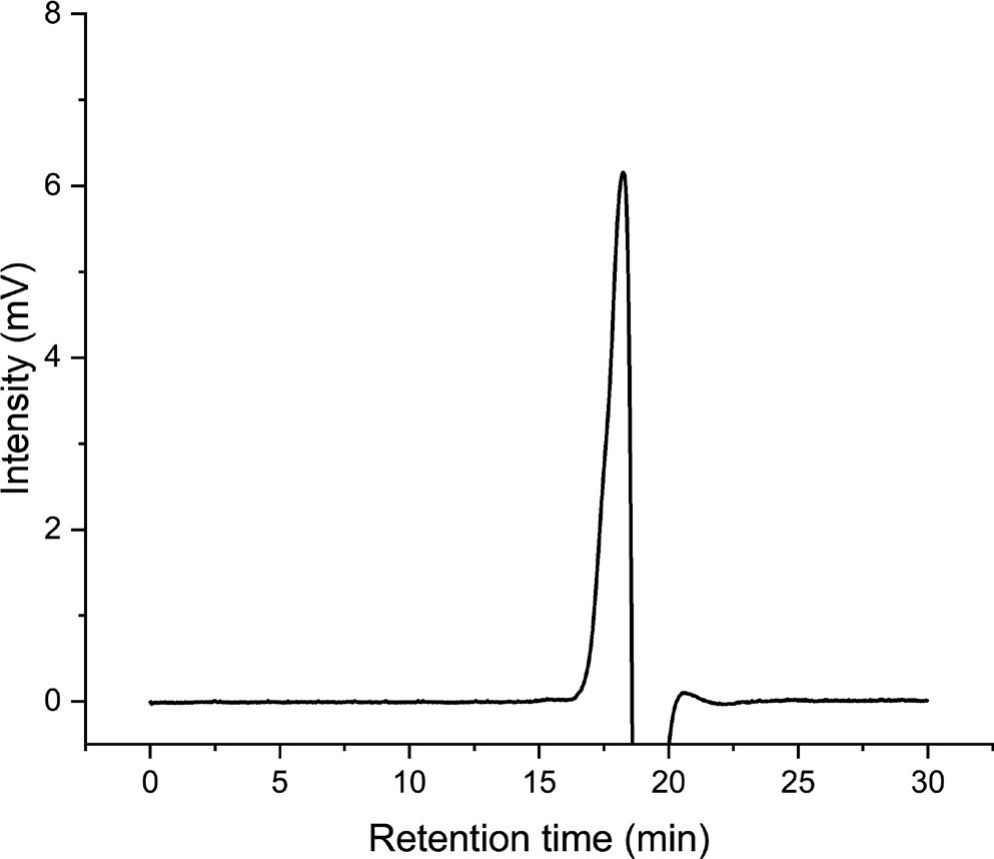
High‐performance gel permeation chromatography (HPGPC) spectrum of RYRO1

The molecular weight of RYRO1 was determined by HPGPC (Figure 7). Based on the log Mw and elution time of the dextran standards, the standard curve equation was calculated as logMw = −0.4556 t + 11.04 (*R* ^2^ = 0.9967). The retention time of the RYRO1 peak was 18.193 min, and the weight average molecular weight was calculated to be 874 Da. The theoretical degree of polymerization (DP) of RYRO1 is about 4.86 based on the molecular weight of glucose (180 Da).

#### 3.3.4 FT‐IR spectrum

**FIGURE 8:**
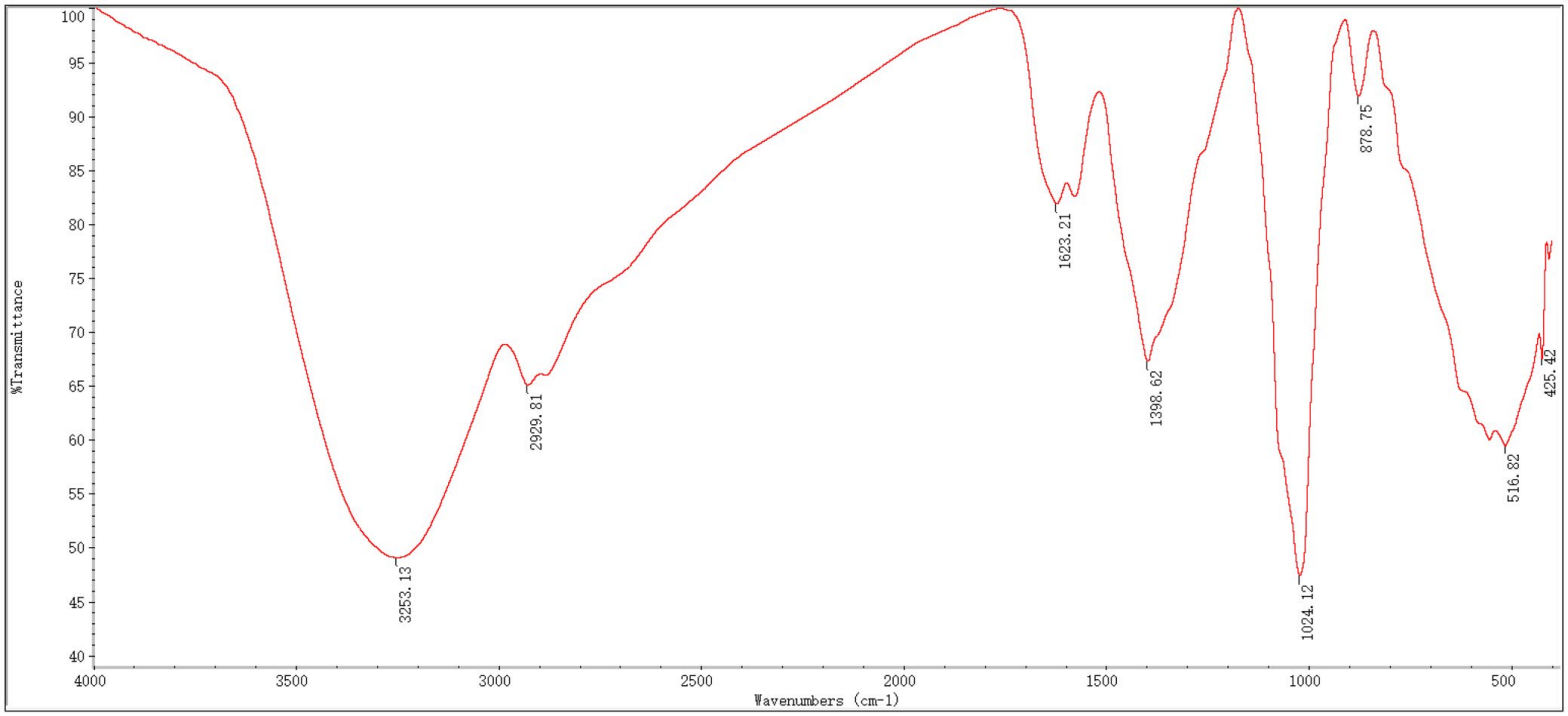
FT‐IR spectra of RYRO1

FT‐IR spectroscopy is a tool commonly used to characterize the functional groups of carbohydrates (He et al., 2021; Zhao et al., 2020). As shown in Figure 8, RYRO1 has eight absorption bands, and the broad‐stretching intense peak near 3253 cm^–1^ is assigned to the stretching vibration of the hydroxyl group (He et al., 2016). A weak‐stretching band at 2929 cm^–1^ is ascribed to the C–H‐stretching vibration (You et al., 2021). The peak at 1024 cm^–1^ is the characteristic absorption peak of asymmetric stretching vibrations of the ether bond on the sugar ring, indicating that there are pyranose units in RYRO1 (He et al., 2021; You et al., 2021). The signal at 1623 cm^–1^ was the characteristic absorption of bonded water, indicating the N–H of primary amine (Xiang et al., 2019). The signal at 1398 cm^–1^ was contributed by the bending vibrations of C–H (Li et al., 2019; Xiang et al., 2019). Moreover, the pyranoside ring absorption band was observed at 878 cm^–1^ (C–H ring vibration) (Nejatzadeh‐Barandozi & Enferadi, 2012) and indicated a β‐glycosidic linkage (Mohammed et al., 2020).

#### 3.3.5 NMR

**FIGURE 9:**
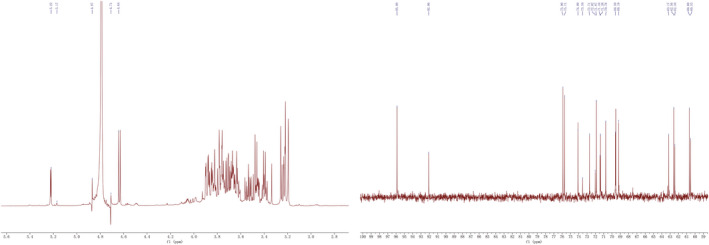
(a) ^1^H‐NMR spectrum of RYRO1; (b) ^13^C‐NMR spectrum of RYRO1

In ^1^H NMR spectra of oligosaccharides, chemical shifts usually appear in the 1.0–6.0 ppm range. From 4.3 ppm to 5.9 ppm was considered to be associated with anomeric protons. The region of 3.0–4.2 ppm is related to the most protons present on C2–C6 with overlap problems (Agrawal, 1992). While in ^13^C NMR spectra of oligosaccharides, the anomeric carbon signals distributed in the range 90–112 ppm (Agrawal, 1992). The structural characterization of RYRO1 was further elucidated by ^1^H‐NMR and ^13^C‐NMR. Chemical shifts (^1^H) between 4.9 and 5.5 ppm are α‐anomeric protons, while signals between 4.4 and 4.9 ppm are assigned to β‐anomeric protons. As seen from ^1^H‐ and ^13^C NMR (Figure 9a,b), the chemical shifts of RYRO1 are distributed in a narrow range from 3.0 to 5.4 ppm, and 60 to 100 ppm, respectively, which indicated the presence of α‐ and β‐configurations in RYRO1. It is inferred from the previous report that the apparent chemical shift signal located at 5.22 ppm indicates the presence of an α‐D‐Glc*p* unit in RYRO1 (Matulova et al., 2011). The signal at 5.17 ppm was assigned to an α‐D‐Man*p* unit (Chen et al., 2021). As shown in ^1^H‐NMR, RYRO1 had anomeric proton signals at 4.87, 4.71, and 4.6 ppm, indicating the presence of β configuration, and they were designated as β‐D‐Gal, β‐D‐Man, and β‐D‐Glc, respectively (Xing et al., 2015; Zhao et al., 2020). In the ^13^C‐NMR spectrum of RYRO1, the shifts at 95.88 correspond to C1 of the β‐D‐Glc*p* reducing units, and the anomeric carbon signal at 92.05 is attributed to C1 of the terminal of α‐D‐Glc*p* (Paredes et al., 2018). Signals at 80–55 ppm related to C2–C6 from the glycosidic ring. Besides, in the ^13^C‐NMR spectrum of RYRO1, there is no significant signal at 80–90 ppm, indicating that RYRO1 does not contain 1 → 3 glucosidic bonds (Jia et al., 2021).

### 3.4 *SEM* analysis

**FIGURE 10:**
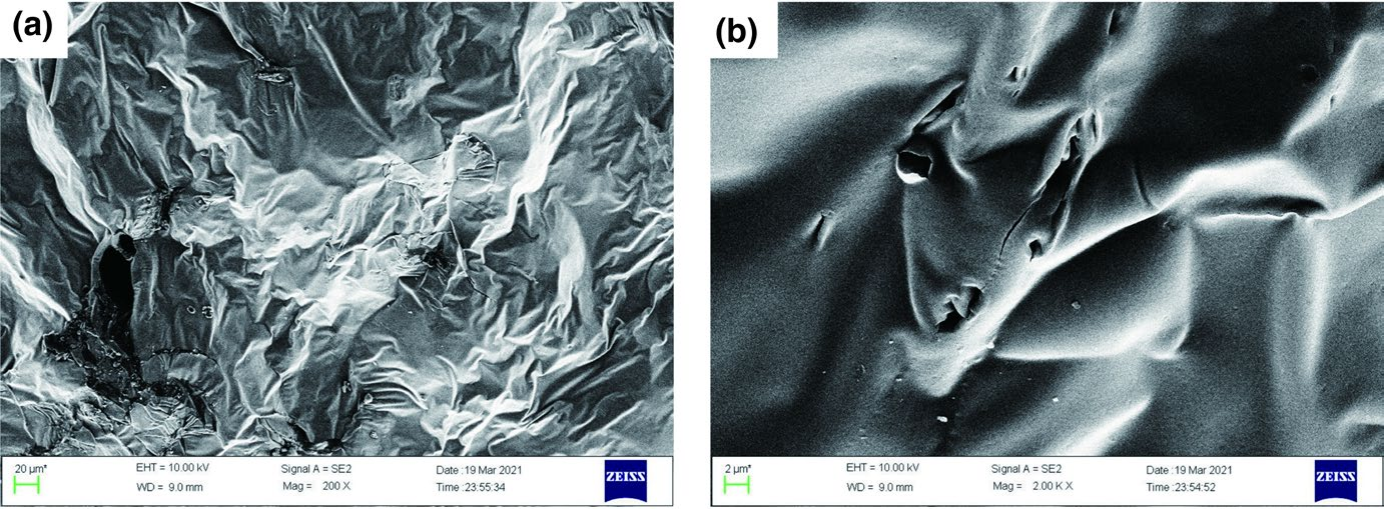
Scanning electron microscopy images of RYRO1 (a: 200×, b: 2000×)

*SEM* has been used to effectively illustrate the surface microstructure of polysaccharides and oligosaccharides (Chaari et al., 2016; Wang et al., 2016). In the present study, the microstructure of RYRO1 was examined by *SEM* (Figure 10). The *SEM* image of RYRO1 at 200 magnification shows that it has a folded paper‐like surface. The image at 2000 magnification shows that its surface is relatively intact and smooth, but with some folds and holes. This may be due to the low degree of branching and short chain structures of RYRO1 (He et al., 2021).

### 3.5 Antioxidant activity

#### 3.5.1 DPPH radical scavenging activity

**FIGURE 11:**
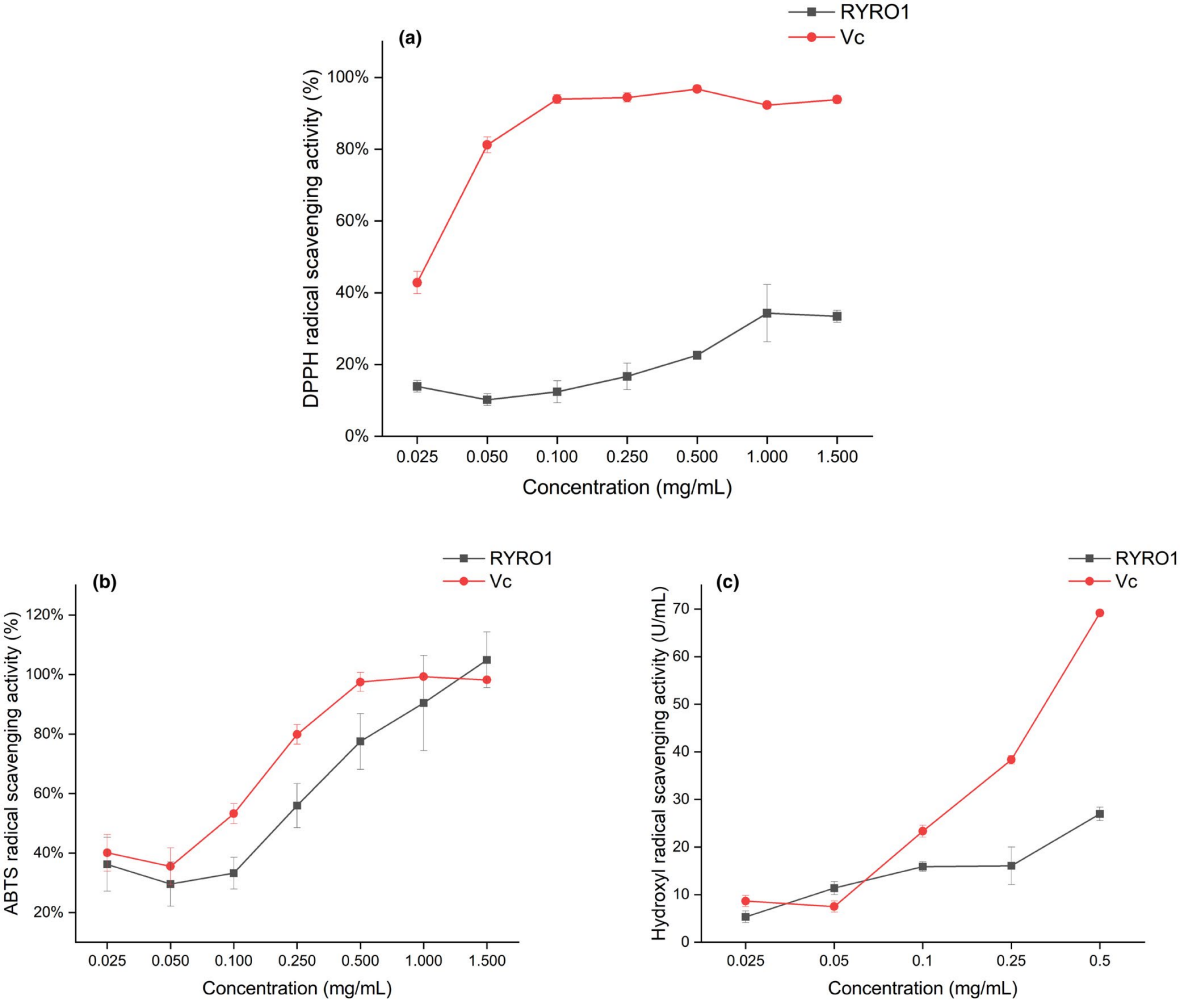
Antioxidant capacity of RYRO1 in vitro, (a) DPPH scavenging; (b) ABTS•+ scavenging; (c) Hydroxyl free radical scavenging

DPPH is a colored, remarkably stable free radical at room temperature and is often used to assess the scavenging capacity of antioxidants (Foti, 2015). The scavenging ability of RYRO1 and Vc for DPPH was measured, and the results are shown in Figure 11a. Overall, the ability of RYRO1 to scavenge DPPH was relatively weak. The scavenging effect of RYRO1 on DPPH was concentration dependent and reached a maximum inhibitory capacity of 34.29% at a concentration of 1 mg/ml, after which there was no further increase. As a positive control, ascorbic acid reached a strong DPPH scavenging capacity at a very low concentration (0.1 mg/ml) (Liu et al., 2019).

#### 3.5.2 ABTS radical scavenging activity

Figure 11b shows the ability of RYRO1 and ascorbic acid to scavenge ABTS radicals. The total antioxidant capacity of a compound is usually estimated in terms of its ability to scavenge ABTS•+. In the range 0.025–1.5 mg/ml, the ability of RYRO1 to scavenge ABTS•+ was concentration dependent. The scavenging capacity of both compounds for ABTS continued to increase overall at the concentrations tested, but ascorbic acid reached maximum scavenging capacity before RYRO1. The ABTS radical scavenging capacity of RYRO1 was observed to be greater than 100% at a concentration of 1.5 mg/ml, 104.91%, which is of course due to the presence of errors. At the same concentration, the scavenging capacity of ascorbic acid was 98.19%. This result indicates that at this concentration, the scavenging capacity of RYRO1 for ABTS is comparable to that of Vc. The scavenging capacity of RYRO1 for ABTS radicals increased roughly linearly in the interval from 0.1 to 1.5 mg/ml. However, the scavenging ability of RYRO1 for ABTS radicals was slightly weaker than that of ascorbic acid when the concentration was below 1.5 mg/ml.

#### 3.5.3 Hydroxyl free radical scavenging activity

Hydroxyl radical is a kind of reactive oxygen species that can cause serious damage to the body (Lipinski, 2011). The scavenging ability of RYRO1 and the positive control (Vc) for hydroxyl radicals are shown in Figure 11c. The scavenging ability of both was concentration dependent. The scavenging capacity of ascorbic acid rose sharply with increasing concentration over the concentration interval tested, while the scavenging capacity of RYRO1 rose slowly. When the concentration reached 0.5 mg/ml, the scavenging capacity of RYRO1 and the positive control reached 26.96 U/ml and 69.16 U/ml, respectively. The test results showed that although the hydroxyl radical scavenging ability of RYRO1 tended to increase in the tested concentration interval, it was generally not as strong as that of ascorbic acid.

## 4 CONCLUSION

In this study, low molecular weight oligosaccharides (RYRO1) with a DP of 4.86 containing mainly glucose, mannose, galactose, and glucosamine were isolated from solid fermented RYR after extraction and a series of purifications for the first time. In vitro experiments showed that low concentrations of RYRO1 displayed antioxidant effects. The scavenging ability of RYRO1 for DPPH, ABTS, and hydroxyl radicals was generally concentration dependent. At 1 mg/ml, the scavenging ability of RYRO1 for DPPH and ABTS radicals was 34.29% and 90.41%, respectively. The scavenging ability of RYRO1 for hydroxyl radicals was relatively weak. At 0.5 mg/ml, RYRO1 could only reach 27 U/mL, while the same concentration of ascorbic acid could reach 69 U/mL.

Free radicals such as reactive oxygen species (ROS) are of paramount relevance in intracellular signaling pathways. It is believed that dysregulated ROS signaling contributed to a plethora of human diseases, such as neurological disorders, cardiovascular system diseases, and diabetes (Finkel, 2011; Sies & Jones, 2020). Antioxidants can counter the negative effects of ROS and thereby avoid or eliminate related diseases, and they are abundant in natural resources. The antioxidant effect of polysaccharides is determined by a combination of many factors, such as molecular weight, protein, phenolic substances, and uronic acid content (Wang et al., 2016). This conclusion may also apply to oligosaccharides, as they are all glycans. Previous studies have shown that the antioxidant capacity of polysaccharide conjugates is directly related to their molecular weight and the content of uronic acid (Chen et al., 2004, 2008). RYRO1 does not contain uronic acid, but it has a small heavy average molecular weight. This may be one of the reasons that explain the results of antioxidant activity in this study. Moreover, the relationship between the antioxidant activity of RYRO1 and its structure remains to be further investigated. The results of this study provide a reference for the development of RYR oligosaccharides as natural antioxidants in food and medicine and the extended application of RYR.

## CONFLICT OF INTEREST

The authors declare that they do not have any conflict of interest.

## AUTHOR CONTRIBUTION

**Kai Liu** and **Long Xie:** data curation (equal); investigation (equal); methodology (equal); writing‐original draft (equal). **Huan Gu:** data curation (supporting); investigation (supporting). **Jia Luo:** conceptualization (lead); funding acquisition (lead); project administration (equal); supervision (equal); writing‐review and editing (lead). **Xiaofang Li:** project administration (equal); supervision (equal).

## ETHICS APPROVAL

This study does not involve any human or animal testing.

## INFORMED CONSENT

Written informed consent was obtained from all study participants.
